# Real-Time Driver Drowsiness Detection Using Facial Analysis and Machine Learning Techniques

**DOI:** 10.3390/s25030812

**Published:** 2025-01-29

**Authors:** Siham Essahraui, Ismail Lamaakal, Ikhlas El Hamly, Yassine Maleh, Ibrahim Ouahbi, Khalid El Makkaoui, Mouncef Filali Bouami, Paweł Pławiak, Osama Alfarraj, Ahmed A. Abd El-Latif

**Affiliations:** 1 Multidisciplinary Faculty of Nador, Mohammed Premier University, Oujda 60000, Morocco; siham.essahraui@ieee.org (S.E.); ismail.lamaakal@ieee.org (I.L.); elhamlyikhlas@gmail.com (I.E.H.); i.ouahbi@ump.ac.ma (I.O.); k.elmakkaoui@ump.ac.ma (K.E.M.); m.filalibouami@ump.ac.ma (M.F.B.); 2 Laboratory LaSTI, ENSAK, Sultan Moulay Slimane University, Khouribga 54000, Morocco; 3 Department of Computer Science, Faculty of Computer Science and Telecommunications, Cracow University of Technology, Warszawska 24, 31-155 Krakow, Poland; pawel.plawiak@pk.edu.pl; 4 Institute of Theoretical and Applied Informatics, Polish Academy of Sciences, Bałtycka 5, 44-100 Gliwice, Poland; 5 Computer Science Department, Community College, King Saud University, Riyadh 11437, Saudi Arabia; oalfarraj@ksu.edu.sa; 6 Jadara University Research Center, Jadara University, Irbid 21110, Jordan; ahmedabdellatif@ieee.org; 7 Department of Mathematics and Computer Science, Faculty of Science, Menoufia University, Shebin El-Koom 32511, Egypt

**Keywords:** drowsy driving, drowsiness detection, computer vision, facial analysis, machine learning

## Abstract

Drowsy driving poses a significant challenge to road safety worldwide, contributing to thousands of accidents and fatalities annually. Despite advancements in driver drowsiness detection (DDD) systems, many existing methods face limitations such as intrusiveness and delayed reaction times. This research addresses these gaps by leveraging facial analysis and state-of-the-art machine learning techniques to develop a real-time, non-intrusive DDD system. A distinctive aspect of this research is its systematic assessment of various machine and deep learning algorithms across three pivotal public datasets, the NTHUDDD, YawDD, and UTA-RLDD, known for their widespread use in drowsiness detection studies. Our evaluation covered techniques including the K-Nearest Neighbors (KNNs), support vector machines (SVMs), convolutional neural networks (CNNs), and advanced computer vision (CV) models such as YOLOv5, YOLOv8, and Faster R-CNN. Notably, the KNNs classifier reported the highest accuracy of 98.89%, a precision of 99.27%, and an F1 score of 98.86% on the UTA-RLDD. Among the CV methods, YOLOv5 and YOLOv8 demonstrated exceptional performance, achieving 100% precision and recall with mAP@0.5 values of 99.5% on the UTA-RLDD. In contrast, Faster R-CNN showed an accuracy of 81.0% and a precision of 63.4% on the same dataset. These results demonstrate the potential of our system to significantly enhance road safety by providing proactive alerts in real time.

## 1. Introduction

Drowsy driving poses a significant global road safety risk, contributing annually to a distressing number of fatalities and severe injuries. Major road safety authorities such as the National Highway Traffic Safety Administration (NHTSA) and the World Health Organization (WHO) underscore the urgency of addressing this preventable issue [1]. In the United States alone, drowsy driving is responsible for approximately 83,000 crashes, 37,000 injuries, and 900 deaths each year [2]. On a global scale, drowsy driving significantly impacts the annual road death toll, highlighting the critical need for effective detection and prevention strategies [3].

In response, advancements in technology have led to the development of DDD systems [4,5,6,7] that use various techniques to assess driver alertness. These include physiological signal analysis involving the use of sensors to monitor indicators such as the brain, heart, eye, and muscle activity, as well as breathing patterns [8,9]. Driving pattern analysis assesses metrics like the speed, steering wheel movements, and lane positioning, referred to as vehicle measures, to gauge alertness [10,11]. Facial feature analysis—using imaging technologies—evaluates visual signs of drowsiness such as the head positioning, eye closure duration, and yawning frequency, focusing on behavioral changes [12,13,14].

Alongside these individual techniques, multimodal systems [15] stand out by combining these techniques, enhancing both accuracy and reliability in detecting drowsiness. They integrate multiple data sources to form a comprehensive profile of a driver’s alertness, effectively identifying subtle signs of fatigue that single-system approaches might miss [16,17,18,19].

Despite the proven effectiveness of physiological and driving pattern methods, their application is often hampered by the need for intrusive equipment and because they provide warnings only after drowsiness symptoms are evident. On the other hand, facial analysis techniques offer several advantages. They are non-intrusive, require minimal setup, and facilitate real-time drowsiness detection. This makes facial analysis not only a proactive approach but also potentially more acceptable and practical for widespread use.

The primary goal of this paper was to evaluate the effectiveness of various CV [20,21,22] and machine learning (ML) techniques [23,24,25,26] in detecting driver drowsiness through facial analysis. We employed three publicly available datasets—the NTHUDD, YawDD, and UTA-RLDD—to perform a comprehensive analysis of the facial indicators of drowsiness, such as yawning and head movements. By contrasting these diverse approaches, the research aimed to identify the most effective methods for improving the real-time detection of driver drowsiness, thereby enhancing road safety and potentially reducing the number of accidents attributed to drowsy driving.

This paper is systematically structured into distinct sections, each dedicated to exploring a specific aspect of the investigation. It begins with a comprehensive literature review that lays the foundation for our research in Section 2. Section 3 provides an overview of the investigative steps and the methods employed. Subsequently, the research findings are presented alongside a comparative analysis, with a benchmarking of the results against prior studies in the field to evaluate the efficacy of different methods in Section 4. Ultimately, we present a summary of our investigation and future work in the Section 5.

## 2. Related Work

This section provides a review of recent research on driver fatigue detection and recognition, specifically emphasizing the analysis of physiological and psychological behaviors, as well as image analysis techniques. These studies utilize advanced deep learning (DL) and ML methodologies to enhance DDD systems, thereby improving road safety.

Peivandi et al. [27] developed a sophisticated DL framework to detect multi-level driver fatigue using physiological signals, particularly EEG, ECG, and EMG signals. The study created a comprehensive multi-level fatigue classification model integrating Generative Adversarial Networks (GANs) and CNNs. The data comprised physiological recordings from drivers under simulated conditions, meticulously validated beyond self-reported measures. The multi-level classification addressed different stages of fatigue, enhancing the model’s practical utility in real-time applications. The model demonstrated remarkable accuracies of 96.8%, 95.1%, and 89.1% across two-level, three-level, and five-level fatigue scenarios, respectively.

Wang et al. [28] developed a novel multi-sensor fusion methodology for real-time fatigue driving recognition, utilizing EEG and ECG signals to monitor physiological changes. The method included visual assessments through in-vehicle and external cameras to monitor the driving behavior and vehicle position, enhancing the accuracy of driver-state assessments. This approach utilized ML algorithms to assess and categorize driving states, offering significant improvements in detecting and responding to driver fatigue, potentially increasing road safety. This technique achieved a remarkable accuracy rate of 96% in identifying fatigue states.

Jiao et al. [29] conducted a comprehensive study on driver fatigue detection using the heart rate variability (HRV) and electrodermal activity (EDA), integrating ML methodologies to improve real-time fatigue identification. The research successfully employed a Light Gradient Boosting Machine for binary classification, attaining a notable 88.7% accuracy.

Chui et al. [30] developed a novel approach for identifying driver drowsiness and stress by applying a deep multiple-kernel learning support vector machine (D-MKL-SVM), optimized by a multiple-objective genetic algorithm (MOGA). Their approach utilizes ECG signals to assess stress and tiredness levels in drivers, with an average sensitivity of 99% and specificity of 98.3% for drowsiness detection. The model achieved a sensitivity of 98.7% and a specificity of 98.4% for stress detection.

Shang et al. [31] investigated the relationship between driver fatigue and psychological conditions by creating a non-invasive technique to concurrently assess a driver’s emotional and fatigue states. Their innovative method combined facial feature analysis with time series data to provide a comprehensive assessment of the driver’s state. By employing an enhanced lightweight RM-Xception convolutional neural network, they attained an accuracy of 73.32% in emotion identification on the Fer2013 dataset. The fatigue detection approach employed dual-threshold methodologies to assess the eye closure and yawn frequency, significantly improving the system’s forecasting accuracy.

Chand and Karthikeyan [32] proposed an innovative DDD system leveraging a CNN and emotion analysis to enhance road safety. Their model integrates a Driver Emotion Detection Classifier (DEDC) to monitor the driver mentality alongside drowsiness, categorizing behaviors into states like fatigue, recklessness, and emotions such as anger or happiness. The system utilizes real-time data from facial recognition and vehicle dynamics (e.g., the RPM, speed) for analysis. The DDD dataset was employed for detecting fatigue, and the extended Cohn–Kanade dataset (CK+) was used for training the emotion analysis.The model achieved an accuracy of 93%.

Nasri et al. [33] developed a DDD system using CNNs and the Viola-Jones algorithm. They used the UTA-RLDD for training and testing, achieving 96% accuracy using custom CNN architectures, emphasizing a balance between complexity and the model accuracy.

Ahmed et al. [34] developed a CNN-based model to detect driver drowsiness by analyzing eye states and facial expressions with a dataset comprising 2900 images categorized into four classes: open, closed, yawning, and no yawning. The model achieved an accuracy of 97%, with a precision, recall, and F1 score of 99%. The study also employed a transfer learning-based VGG16 model, which yielded a lower accuracy of 74%.

Krishna et al. [35] introduced a novel DDD framework using YoloV5 for face detection and Vision Transformers (ViTs) for binary image classification. The model was trained on the UTA-RLDD and tested on a custom dataset of 39 participants, demonstrating robustness across various lighting conditions. The ViT architecture achieved 96.2% training and 97.4% validation accuracies, while the system showed an overall testing accuracy of 95.5% on the custom dataset.

While the studies above primarily focused on performing a comprehensive analysis of the entire facial area in each image through ML and CV techniques, other research concentrated on more specific regions of the face, particularly the eyes and mouth. Civik et al. [36] developed a driver fatigue detection system that utilizes two separate CNN models to analyze the eye and mouth regions, trained on the YawDD. The eye model achieved an accuracy of 93.6%, while the mouth model reached 94.5%. The same dataset was tested by He et al. [37] using a two-stage CNN architecture, including a Location Detection Network for feature extraction and a State Recognition Network for fatigue state classification. The State Recognition Network achieved impressive performance, with an accuracy of 93.83% on the validation set.

Other studies, such as that by Rajamohana et al. [38], combined a CNN and Bidirectional Long Short-Term Memory (BiLSTM) to detect drowsiness through eye blink patterns, achieving 94% accuracy. Dey et al. [39] analyzed facial landmarks and utilized SVM classifiers, reaching a peak accuracy of 96.4%. Maheswari et al. [40] employed a CNN to analyze mouth and eye closure states, obtaining 95.67% accuracy under diverse conditions. Mehta et al. [41] developed AD3S, a real-time detection system implemented as an Android app that utilized various ML techniques, achieving around 98% accuracy with bagging classifiers. Additionally, Ahmed et al. [42] proposed an ensemble model with InceptionV3 that achieved a test accuracy of 97.1% on the NTHUDDD dataset. Finally, Zhang et al. [43] introduced a privacy-preserving federated learning framework for drowsiness detection, achieving up to 86% accuracy on the YawDD dataset.

There are also DL-based systems for detecting driver fatigue that have been trained on video sequences, as demonstrated in a study by Fa et al. [44]. They proposed a lightweight Multi-Scale Spatial–Temporal Attention Graph Convolutional Network (MS-STAGCN) that uses skeletal data for drowsiness detection. Evaluated on the NTHUDDD dataset, the model achieved an accuracy of 92.4%.

Majeed et al. [45] developed a deep CNN-based model for detecting driver drowsiness focused on the Mouth Aspect Ratio (MAR), achieving 96.69% accuracy using the YawDD and data augmentation techniques. Bai et al. [46] introduced a two-stream spatial–temporal graph convolutional network (2s-STGCN), capturing spatial and temporal features from facial landmarks, with accuracies of 93.4% and 92.7% on the YawDD and NTHUDDD datasets, respectively. Weng et al. [47] employed a Hierarchical Temporal Deep Belief Network (HTDBN), combining Deep Belief Networks (DBNs) and Hidden Markov Models (HMMs) for drowsiness detection, and tested it on a diverse custom dataset. Phan et al. [48] integrated DL networks with IoT technologies for real-time driver fatigue detection, achieving up to 98% accuracy. Finally, Bekhouche et al. [49] developed a hybrid framework using YOLO for face detection and ResNet-50 for feature extraction, refined by a novel algorithm (FCFS), achieving 86.74% accuracy on the NTHUDDD dataset.

Table 1 provides a comprehensive summary of the reviewed literature, highlighting the facial analysis methods employed for DDD. It outlines the approaches, methodologies, and datasets used, along with the most significant results achieved in each study.

## 3. Methodology

**Figure 1:**
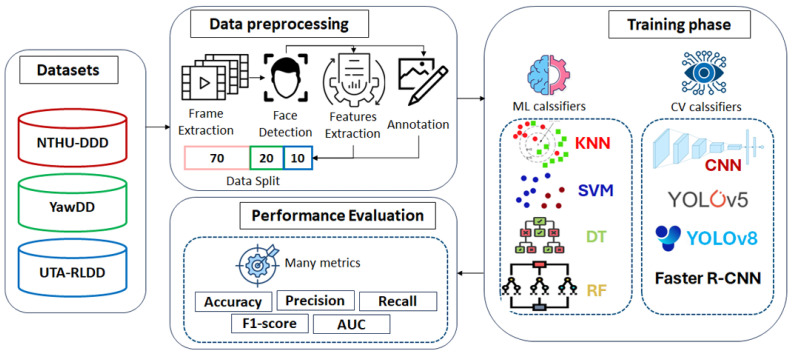
Architecture of the proposed methodology.

This research evaluates ML and CV techniques for DDD, as illustrated in the workflow diagram shown in Figure 1. The methodology initiated with data collection from three primary datasets, the NTHUDDD, YawDD, and UTA-RLDD, focusing on a variety of driving behaviors, such as yawning and regular driving. The data preprocessing phase involved frame extraction, face detection, and feature extraction from video data. These frames were then annotated, and the data were split into training, validation, and testing subsets.

During the training phase, ML classifiers such as the KNNs, SVM, DTs, and RF were employed alongside CV classifiers, including CNNs, YOLOv5, YOLOv8, and Faster R-CNN. To ensure a solid evaluation of the detection systems, the performance of these models was rigorously evaluated using several metrics, including the accuracy, precision, recall, F1 score, and area under the curve (AUC). Further details on these procedures are provided in subsequent sections of the study.

### 3.1. Benchmark Datasets

**Figure 2:**
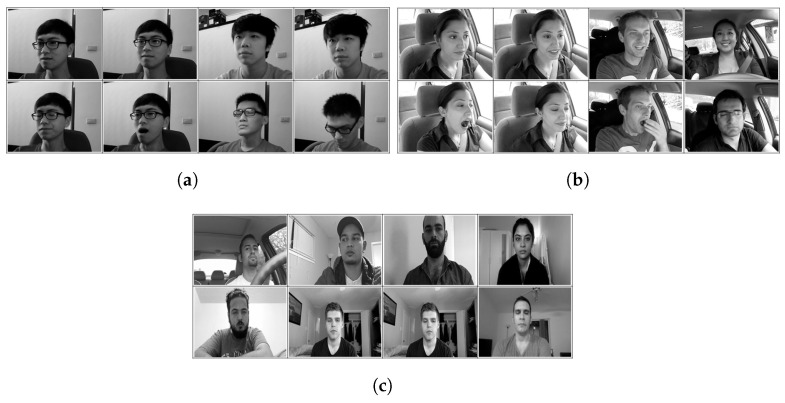
Sample images from three drowsiness detection datasets. (**a**) Sample images from NTHUDDD dataset; (**b**) sample images from YAWDD; (**c**) sample images from UTA-RLDD.

In this assessment, three public datasets of driver drowsiness were used to train and test the ML and CV methods. These were the UTA-RLDD, NTHUDDD, and YawDD (see Figure 2). Each dataset had its own collection method and scenario, annotation mode, dataset size, and facial expressions. This section provides more information about these three datasets.

#### 3.1.1. NTHU Drowsy Driver Detection (NTHUDDD)

The NTHUDDD dataset [53] is publicly available and was collected by the CV Laboratory at the National Tsing Hua University. It consists of 36 infrared video recordings captured under various simulated driving conditions, including normal driving, slow yawning, falling asleep, and laughing out loud, among others. The videos were recorded under both daytime and nighttime lighting conditions, with all scenarios involving simulated fatigue.

#### 3.1.2. Yawning Detection Dataset (YawDD)

The YawDD [55], curated by the Distributed Collaborative Virtual Environments Research Laboratory (DISCOVER Lab) at the University of Ottawa, includes two distinct sub-datasets. The first sub-dataset comprises 322 videos showcasing normal facial expressions, while the second contains 29 videos of drivers yawning. Both sub-datasets feature a diverse group of participants, including male and female drivers of various racial backgrounds, with and without glasses or sunglasses.

#### 3.1.3. UTA Real-Life Drowsiness Dataset (UTA-RLDD)

The UTA-RLDD [52] was developed for the multi-level detection of drowsiness. The primary focus of this dataset is to capture subtle microexpressions indicative of fatigue, rather than only extreme and easily noticeable signs of sleepiness. It includes 60 healthy participants who recorded a total of 30 h of RGB video footage, utilizing their personal phones or webcams to capture facial expressions in real-life scenarios. Due to the physiological and instinctive nature of fatigue-related expressions, the participants found it difficult to artificially replicate the subtle microexpressions associated with sleepiness.

### 3.2. Data Preparation

In this study, we utilized three datasets consisting of video data, from which individual frames were extracted and categorized into two classes: ‘drowsy’ and ‘non-drowsy’. This step provided the foundation for subsequent processing tailored to the needs of different ML techniques.

- For ML models, such as the KNNs and SVMs, we followed a structured preprocessing pipeline that began with facial region detection and feature extraction. Using the Haar Cascade Classifier [56], we accurately identified and isolated facial regions within each frame. This step was crucial in narrowing the focus to areas of interest associated with driver drowsiness. Once the facial regions were detected, a Histogram of Oriented Gradients (HOG) was applied to extract essential features by capturing information about the texture and shape. These features provided critical input for the classification models, enabling them to differentiate between drowsy and non-drowsy states effectively.
- For DL-based object detection models, such as YOLO and Faster R-CNN, a different data preparation approach was employed. The frames were meticulously labeled with bounding boxes around key regions of interest, such as the eyes and other facial features. These annotations were formatted specifically for each model: the YOLO format was used for YOLO-based models, while XML annotations were prepared for Faster R-CNN. This labeling process ensured that the models could accurately learn to detect relevant features and patterns associated with drowsiness.

By isolating critical regions and extracting meaningful features, we enhanced the models’ ability to analyze the data effectively while reducing the computational overhead.

### 3.3. ML Models

This section offers a brief overview of the ML algorithms used in this study. It highlights the key parameters (see Table 2), underlying mathematical principles, and implementation details of each method, emphasizing their importance in the classification process.

#### 3.3.1. K-Nearest Neighbors

The KNNs algorithm [57] primarily depends on the n_neighbors parameter, determining the number *k* of the nearest neighbors to consider for classification. In this case, the optimal value of *k* was determined to be 1 by testing various values to identify the one that achieved the highest accuracy on the test set. The classification of a data point, *x*, is mathematically described as follows:

(1) $$\mathrm{Class}\left(x\right)=\mathrm{mode}(\mathrm{Class}\left(x_{1}\right),\mathrm{Class}\left(x_{2}\right),\ldots ,\mathrm{Class}\left(x_{k}\right))$$

where $x_{1},x_{2},\ldots ,x_{k}$ are the *k* closest points to *x*. The predicted class for *x* is determined by the mode, which is the most frequent class among its *k* nearest neighbors. This method capitalizes on the local structure of the data by leveraging the labels of the nearest data points to infer the class of *x*.

#### 3.3.2. Support Vector Machines

The SVM [58] is a supervised learning model used for classification, depending on the kernel function and the regularization parameter C. It is trained using a linear kernel that maps data into a higher dimensional space where a hyperplane can separate the classes. The optimization problem for finding the optimal hyperplane is

(2) $$\min_{w,b}\frac{1}{2}{∥w∥}^{2}\;\mathrm{subject}\;\mathrm{to}\;y_{i}\left(w\;\cdot \;x_{i}+b\right)\geq 1,\;\forall i.$$

where *w* is the weight vector, *b* is the bias term, and $y_{i}$ are the class labels. The parameter *C* controls the trade-off between maximizing the margin and minimizing misclassification errors. A typical value for *C* is 1.0.

#### 3.3.3. Decision Tree

The DT algorithm [59] is a model used for classification and regression tasks, where data are split into subsets based on feature values. The splitting criteria, such as the Gini impurity or entropy, measure the quality of the splits. The process is recursive, continuing until a stopping condition is met. In this case, the random_state parameter was set to 42 to ensure the reproducibility of the results. The impurity of a dataset, *S*, is defined by the entropy as follows:

(3) $$\mathrm{Entropy}\left(S\right)=-\sum_{i=1}^{k}p_{i}\log_{2}p_{i}$$

Here, $p_{i}$ is the probability of class *i* in *S*. Predictions are made by traversing the tree from the root to a leaf, where the assigned class or value is determined.

#### 3.3.4. Random Forest

The RF [60] is an ensemble learning method that combines the predictions of multiple DTs to make a final decision. Instead of relying on a single tree, it builds several trees during training, each using a random subset of the data and features. This randomness helps the model generalize better and avoid overfitting. In this case, we set the n_estimators parameter to 100 to specify the total number of trees and the random_state to 42 to ensure consistent results each time we ran the model.

To predict the class for a data point, *x*, the model takes the majority vote from all the trees, calculated as

(4) $$\mathrm{Class}\left(x\right)=\frac{1}{N}\sum_{i=1}^{N}{\mathrm{Class}}_{i}\left(x\right)$$

Here, the following apply:

- *N* is the total number of trees in the forest.
- $Class_{i}\left(x\right)$ is the predicted class of the *i*th tree.

### 3.4. CV Algorithms

This section examines prominent CV techniques, including CNNs, YOLO variants, and Faster R-CNN, commonly applied to tasks such as object detection and classification.

#### 3.4.1. Convolution Neural Network

CNNs [61] are widely used DL models designed for efficient feature extraction and pattern recognition from spatial data, particularly images. In our case, this network architecture (see Table 3) begins with an input layer designed to accommodate the spatial and channel dimensions of the input data, followed by a series of convolutional layers interleaved with batch normalization to ensure faster convergence and better generalization. Residual connections are incorporated to enable feature reuse and stabilize the learning process in deeper layers, defined mathematically as

(5) Residual(x,y)=x+y

The convolution operation, fundamental to feature extraction, applies a kernel, *k*, over the input matrix *x* as

(6) $$\mathrm{Conv}\left(x,k\right)=\sum_{i=1}^{m}\sum_{j=1}^{n}x\left[i,j\right]\;\cdot \;k\left[i,j\right]$$

The ELU activation introduces non-linearity, defined by

(7) $$\mathrm{ELU}\left(x\right)=\left\{\begin{array}{cc} x, & \mathrm{if}\;x>0\\ \alpha (\exp(x)-1), & \mathrm{if}\;x\leq 0 \end{array}\right.$$

where α>0 controls the behavior for negative inputs. To reduce the spatial dimensions, the network employs the MaxPooling2D and GlobalAveragePooling2D layers, the latter being defined as

(8) $$\mathrm{GlobalAvgPool}\left(x\right)=\frac{1}{m\times n}\sum_{i=1}^{m}\sum_{j=1}^{n}x\left[i,j\right]$$

The architecture concludes with fully connected dense layers and a sigmoid activation for binary classification, offering an effective balance of spatial feature extraction, efficient dimensionality reduction, and high-level feature learning.

#### 3.4.2. YOLOv5

YOLO (You Only Look Once) [62] is a DL model family renowned for real-time object detection, balancing speed and accuracy. The YOLOv5s variant, utilized in this study, operates on 640 × 640 pixel input images with a batch size of 16 and is trained over 50 epochs. We fine-tuned this model for binary classification, enabling it to distinguish between cheating and non-cheating behaviors.

For each detected instance, the YOLOv5s model predicts the bounding box coordinates (x,y,w,h), where *x* and *y* denote the center, and *w* and *h* represent the width and height. The confidence score for the bounding box is computed as

(9) $$P_{\mathrm{box}}=\sigma \left(t_{x}\right)\;\cdot \;\sigma \left(t_{y}\right)\;\cdot \;\exp\left(t_{w}\right)\;\cdot \;\exp\left(t_{h}\right)$$

where $\sigma (t_{x})$ and $\sigma (t_{y})$ are the normalized center offsets, and $\exp(t_{w})$ and $\exp(t_{h})$ represent the predicted width and height in exponential space, ensuring non-negative values.

#### 3.4.3. YOLOv8

YOLO has made remarkable strides in its evolution, with YOLOv8 [63] setting new standards in object detection performance. The YOLOv8n model was fine-tuned over 50 training epochs so that it could perform a binary classification task and tell the difference between two target classes correctly. The training process optimizes the model’s performance by minimizing the loss function, which combines the localization, confidence, and classification errors. The following localization loss function determines the object detection performance:

(10) $$L_{\mathrm{loc}}=\sum_{i=1}^{N}\mathrm{CIoU}\left(b_{i},{\hat{b}}_{i}\right)$$

where $b_{i}$ represents the predicted bounding box, ${\hat{b}}_{i}$ is the ground truth bounding box, and CIoU denotes the Complete Intersection over Union, a metric that considers both the overlap and distance between bounding boxes.

#### 3.4.4. Faster R-CNN

Faster R-CNN [64] is a state-of-the-art object detection model that combines region proposal and classification into a unified architecture. In this study, a Faster R-CNN model with a ResNet-50 backbone [65] and Feature Pyramid Network (FPN) [66] was trained on a custom dataset. The training process was conducted over 50 epochs with a batch size of 8.

The model leverages a Region Proposal Network (RPN) [67] to generate candidate object regions, followed by a classification head to predict object categories and refine bounding box coordinates. The optimization process minimizes a multi-task loss function, defined as

(11) $$L=L_{\mathrm{cls}}+L_{\mathrm{bbox}}$$

where $L_{\mathrm{cls}}$ represents the classification loss and $L_{\mathrm{bbox}}$ denotes the bounding box regression loss.

## 4. Experimentation and Results

This section provides an overview of the diverse evaluation metrics employed in our investigation, alongside a detailed examination of the findings from ML and CV models.

### 4.1. Evaluation Metrics and Measures

During the training and testing stages, we evaluated the ML and CV models’ performances using commonly employed metrics. These measures included confusion matrices, from which many metrics were derived, like the accuracy, precision, recall, and F1 score, and the ROC AUC metric, measuring a model’s ability to differentiate between drowsy and awake states, with higher scores indicating a superior discrimination capability. Finally, we used the mAP metric to evaluate our object detection models. Equations (12)–(16) present these metrics.

(12) $$Accuracy=\frac{TP+TN}{TP+TN+FP+FN}$$

(13) $$Precision=\frac{TP}{TP+FP}$$

(14) $$Recall=\frac{TP}{TP+FN}$$

(15) $$F1-score=2*\frac{P*R}{P+R}$$

(16) $$mAP=\frac{1}{N}\sum_{i=1}^{N}AP_{i}$$

In a confusion matrix, true positives (TPs) stand for true positive samples, true negatives (TNs) for true negative samples, false positives (FPs) for false positive samples, and false negatives (FNs) for false negative samples. N is the number of classes, and $AP_{i}$ is the AP of class i.

### 4.2. Performance of ML Approaches

Table 4 provides a comparative analysis of three distinct datasets, the NTHUDDD, YawDD, and UTA-RLDD, across four classifiers: the KNNs, SVM, DTs, and RF. The KNNs classifier performed remarkably well, particularly on the UTA-RLDD, achieving the highest test accuracy of 98.89% and a recall of 98.12%. It also secured the best F1 score of 98.86% and the highest AUC of 98.79%. The SVM classifier demonstrated commendable performance on the same dataset, with a test accuracy of 97.76% and a precision of 97.45%. The RF classifier was noted for its strong precision of 99.58% on the same dataset. In the NTHUDDD dataset, the KNNs attained a test accuracy of 95.72% and a recall of 96.31%. Furthermore, it achieved a precision of 95.34% and an F1 score of 95.72%, indicating a balanced performance across several measures. In contrast, the DT classifier performed poorly, especially on the YawDD, where it achieved the lowest scores, with a test accuracy of just 67.14% and an AUC of 67.08%.

**Figure 3:**
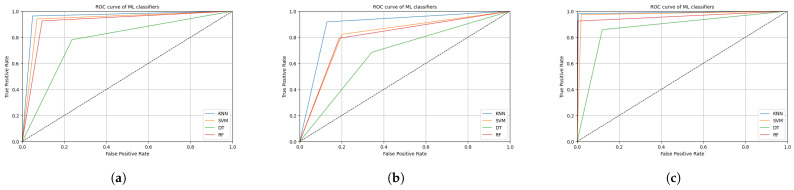
Roc curves for different datasets. (**a**) Roc curve for NTHUDDD dataset; (**b**) Roc curve for YawDD; (**c**) Roc curve for UTA-RLDD.

Figure 3 illustrates the ROC curves for the four ML classifiers—the KNNs, SVM, DTs, and RF—for our three datasets. The ROC curve for the UTA-RLDD demonstrates that all classifiers, especially the SVM and KNNs, attained near-perfect performance, with curves closely hugging the upper left corner, indicating high true positive rates and minimal false positive rates. Different classifiers worked better or worse on the YawDD. The RF and SVM both did a good job, but they were a little farther from the best top-left corner than they were on the NTHUDDD dataset. The NTHUDDD dataset presented a similar scenario where the KNNs and SVM maintained better performance over the RF and DTs, showing their resilience across diverse datasets. Overall, the KNNs and SVM were better at telling the difference between classes across all datasets. However, the RF and DTs showed differences, having more trouble with the YawDD and NTHUDDD dataset in particular.

**Figure 4:**
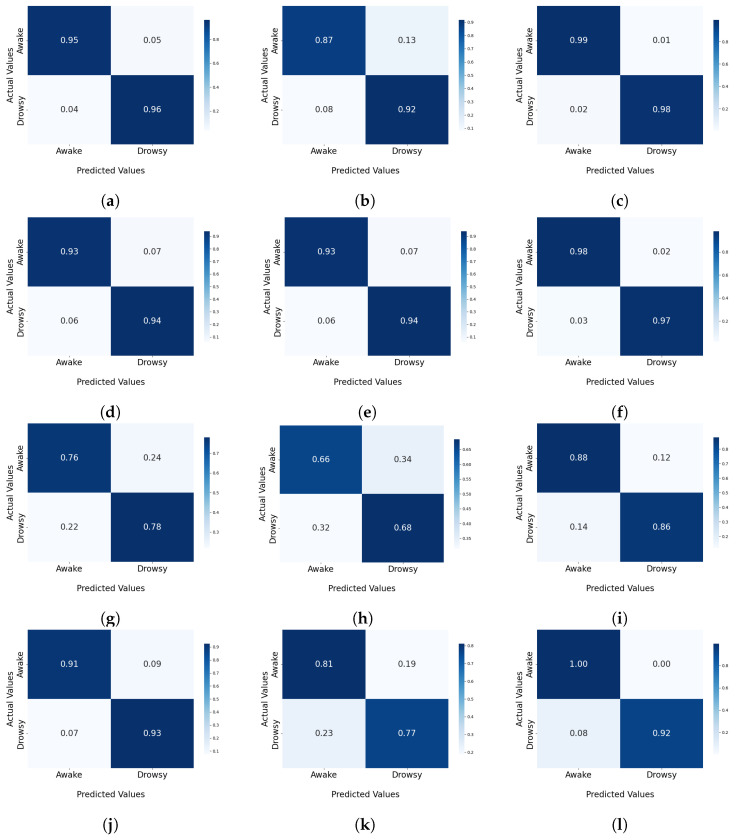
Confusion matrix for ML algorithms across three datasets. (**a**) kNNs for Dataset 1; (**b**) kNNs for Dataset 2; (**c**) kNNs for Dataset 3; (**d**) SVM for Dataset 1; (**e**) SVM for Dataset 2; (**f**) SVM for Dataset 3; (**g**) DT for Dataset 1; (**h**) DT for Dataset 2; (**i**) DT for Dataset 3; (**j**) RF for Dataset 1; (**k**) RF for Dataset 2; (**l**) RF for Dataset 3.

The confusion matrices presented in Figure 4 provide a comprehensive comparison of the ML methods applied across the three diverse datasets. For the SVM and KNNs techniques, there was significant variability in their performance outcomes. These techniques attained near-optimal classification on the UTARLDD with TP and TN rates approaching 99% and minimal FPs and FNs. Conversely, the SVM performance on the YawDD showed higher numbers of FPs and FNs, with a TP rate of approximately 94% and TN rate of around 93%, indicating significant challenges in generalization and specificity for yawning detection. Focusing on the NTHUDDD dataset, the kNN algorithm demonstrated robust performance, achieving TP and TN rates of 95%, indicating proficient classification skills. The SVM demonstrated a high TP rate of 94% and a TN rate of 93%, along with low FP and FN rates of around 7%, highlighting its efficacy in detecting driver sleepiness.

The DT model showed a TP rate of 78% and a TN rate of 76% on the NTHUDDD dataset, which slightly decreased on the YawDD to 68% TPs and 66% TNs but improved on the UTARLDDD with a TP rate of 86% and a good TN rate of 88%. The RF method achieved the best result with the UTA-RLDD, maintaining a high TP rate of 92% and a perfect TN rate of 100%.

### 4.3. Performance of CV Approaches

The performance metrics of the CNN across three distinct datasets—the NTHUDDD, YawDD, and UTA-RLDD—reflected its high efficacy and adaptation to varied testing conditions (see Table 5).

- The NTHUDDD dataset demonstrated robust training and test accuracies at 99.31% and 98.22%, respectively, with the precision and recall also exceeding 98%, indicating exceptional proficiency in detecting sleepiness in controlled environments.
- There were some challenges with the YawDD, as shown by the lower test accuracy of 93.31% and the F1 score of 93.31%. This means that the model may not be able to generalize as well when it comes to detecting yawning.
- The UTA-RLDD illustrated near-perfect model performance, achieving a test accuracy of 99.97% and a precision of 100%.

**Figure 5:**
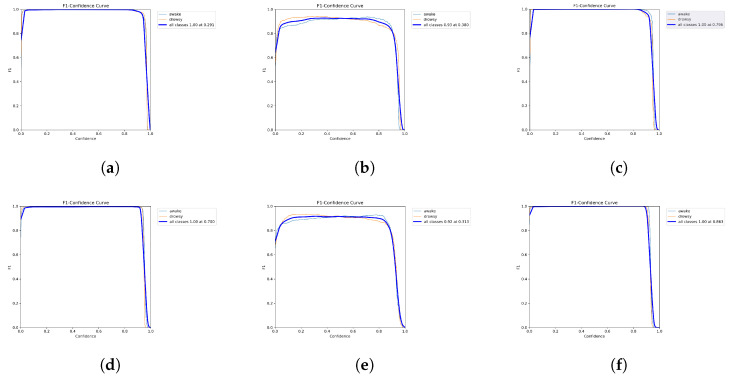
F1 curve for YOLOv5 (**a**–**c**) and YOLOv8 (**d**–**f**) techniques. (**a**) F1 curve for NTHUDDD dataset; (**b**) F1 curve for YawDD; (**c**) F1 curve for UTA-RLDD; (**d**) F1 curve for NTHUDDD dataset; (**e**) F1 curve for YawDD; (**f**) F1 curve for UTA-RLDD.

Figure 5 illustrates the F1 score training curves for YOLOv5 and YOLOv8, which were evaluated across three separate datasets—the NTHUDDD, YawDD, and UTA-RLDD—emphasizing their performance throughout the training process.

On the NTHUDDD dataset, both YOLOv5 and YOLOv8 exhibited remarkable learning capabilities, achieving an F1 score of 1.00 at confidence thresholds of approximately 0.291 and 0.7, respectively, signifying their robust capacity to identify drowsiness in simulated driving scenarios. However, the YawDD presented more challenges, with YOLOv5 and YOLOv8 achieving lower F1 scores of 0.92 and 0.93 at confidence thresholds of approximately 0.313 and 0.380, respectively. These results indicate a strong detection capacity, though somewhat less consistent compared to the performance on the NTHUDDD dataset.

Using the UTA-RLDD, both models achieved nearly perfect results, with F1 scores of 1.00 at confidence levels of approximately 0.796 for YOLOv5 and 0.863 for YOLOv8. This demonstrates their exceptional ability to detect drowsiness in a range of real-world scenarios.

**Figure 6:**
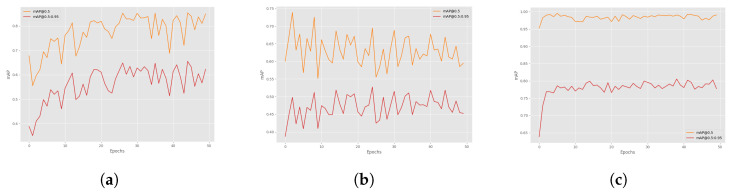
mAP graphs for different datasets using Faster R-CNN technique. (**a**) mAP graph for NTHUDDD dataset; (**b**) mAP graph for YawDD; (**c**) mAP graph for UTA-RLDD.

The graphs in Figure 6 show the Mean Average Precision (mAP) of our three datasets: the NTHUDDD, YawDD, and UTA-RLDD. They are shown over 50 epochs to show how the Faster R-CNN model’s accuracy changed at different IoU thresholds.

- The NTHUDDD Dataset (Figure 6a): Two mAP lines are illustrated, one for IoU = 0.5 (mAP@0.5) and another for IoU = 0.5:0.95 (mAP@0.5:0.95). The mAP@0.5 stayed high, averaging around 0.8 during training. This shows that the model could consistently accurately detect drowsiness at a basic intersection over union threshold. On the other hand, the mAP@0.5:0.95, which was lower, showed gradual improvement, which means the model got more accurate at stricter IoU thresholds.
- The YawDD (Figure 6b): The mAP scores for both IoU thresholds exhibited more variability compared to those of the NTHUDDD dataset. The mAP@0.5 averaged about 0.65, which means it had a moderate ability to detect things. This might be because the dataset was diverse, with different yawning expressions and possibly different levels of video quality. The mAP@0.5:0.95 metric was significantly lower, starting just above 0.4 and showing no improvement, suggesting difficulties in attaining high accuracy over stricter IoU thresholds.
- The UTA-RLDD (Figure 6c): This model exhibited the greatest mAP@0.5 scores among the three, nearly reaching 1.0 after the initial epochs, which suggests exceptional model performance in real-world situations. The mAP@0.5:0.95 started out higher than that of the other datasets and kept going up until it stopped around 0.85, which suggests that it could find smaller signs of sleepiness even when the IoU thresholds were very strict.

The graphs show the different problems and how well the detection system worked across several datasets. The UTA-RLDD showed the best performance, which means the model can adapt and make accurate predictions in a wider range of real-world situations. The YawDD mAP’s heterogeneity highlights potential areas for model tuning to enhance the consistency and accuracy.

In the inference phase, several techniques were employed to assess the effectiveness of CV methods, such as the precision, recall, and mAP, at different intersection over union (IoU) thresholds (see Table 6).

The YOLOv5 and YOLOv8 models consistently performed very well across the NTHUDDD dataset, YawDD, and UTA-RLDD, demonstrating high precision, recall, and overall effectiveness. On the NTHUDDD dataset, YOLOv5 achieved a precision of 99.9% and a recall of 100%, with mAP scores of 99.5% at IoU = 0.5 and 97.9% at IoU = 0.5–0.95. YOLOv8 matched this exemplary performance, with a precision of 99.9% and marginally higher mAP scores, demonstrating robust detection capabilities. On the YawDD, both models exhibited robust performance, but with marginally diminished accuracy and mAP scores relative to those of the NTHUDDD dataset. YOLOv5 attained a precision of 90.6%, whereas YOLOv8 recorded 88.2%, with the mAP values indicating efficient detection, though with slightly greater variability in performance. Both models performed exceptionally on the UTA-RLDD; YOLOv5 and YOLOv8 achieved an accuracy of 99.9% and a recall of 100%, demonstrating their effectiveness in real-world scenarios.

In contrast, Faster-RCNN exhibited lower performance across all the datasets. The NTHUDDD dataset showed an accuracy of 63.4% and a recall of 77.7%, notably lower than the performance of the YOLO models. The YawDD further highlighted its limitations, with the accuracy decreasing to 53.7% and the recall to 69%, reflecting challenges in adapting to variations in the dataset characteristics. In the UTA-RLDD, Faster-RCNN demonstrated a significant improvement, with the accuracy increasing to 81.0% and the recall to 84.9%, suggesting better alignment with the dataset’s characteristics for more effective detection. Despite this improvement, Faster-RCNN generally lagged behind the YOLO models in terms of both precision and reliability across diverse detection scenarios.

**Figure 7:**
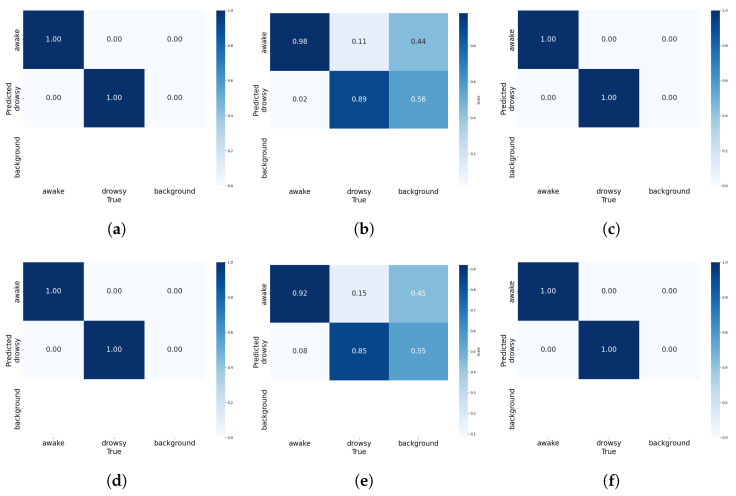
Confusion matrix for CV methods across three datasets. (**a**) YOLOv5 for Dataset 1; (**b**) YOLOv5 for Dataset 2; (**c**) YOLOv5 for Dataset 3; (**d**) YOLOv8 for Dataset 1; (**e**) YOLOv8 for Dataset 2; (**f**) YOLOv8 for Dataset 3.

The confusion matrices displayed for YOLOv5 and YOLOv8 across our three datasets demonstrate the efficacy of these CV models in categorizing ‘awake’ and ‘drowsy’ states, as well as managing ‘background’ classifications (see Figure 7).

On the NTHUDDD dataset and UTA-RLDD, both YOLOv5 and YOLOv8 achieved a perfect classification accuracy, correctly identifying all instances of ‘awake’ and ‘drowsy’ without any errors, demonstrating their resilience in a controlled testing environment. However, on the YawDD, the models exhibited diminished performance; YOLOv5 accurately identified ‘awake’ 98% of the time and ‘drowsy’ 89% of the time, whereas YOLOv8 demonstrated marginally lower accuracy with 92% for ‘awake’ and 85% for ‘drowsy’, with notable misclassification, particularly in distinguishing between ‘drowsy’ and ‘background’.

### 4.4. Discussion

This study presents significant advancements in DDD using cutting-edge ML and CV methodologies. The results demonstrate enhanced accuracy and flexibility across various datasets, highlighting the effectiveness of these approaches. The tests showed that YOLOv5 and YOLOv8 were better at finding drivers who were falling asleep than both Faster R-CNN and regular ML methods. The KNNs was the most accurate of the ML methods on the UTA-RLDD, and the SVM was not far behind, also obtaining impressive performance values. Nevertheless, CV models consistently outperformed ML approaches. YOLOv5 attained the highest accuracy and recall on the UTA-RLDD, demonstrating superior performance, while YOLOv8 delivered similarly high metrics. The CNN demonstrated robust performance. Conversely, Faster R-CNN struggled with significantly lower accuracy and recall, emphasizing its limitations in handling diverse conditions.

In Table 7, we show a full comparison of how accurate our models were across different datasets compared to what other studies have found.

The KNN classifier exhibited a notable increase in accuracy, rising from 83% in the work of Kiashari et al. [68] to 98.89% for the UTA-RLDD, reflecting a considerable advancement in the classification performance. Similarly, the SVM classifier showed robust performance, achieving an accuracy of 97.76% for the UTA-RLDD compared to 94.9% in the study by Maior et al. [69], despite a slight decrease to 81.13% for the YawDD, indicating minor difficulties in adapting to its special characteristics. The DT classifier, which previously achieved an accuracy of 65.41% in the work of Mittal et al. [70], now exhibits improved results, with 87.12% for the UTA-RLDD. The performance of RF classifiers also got better, with the accuracy going from 82.3 percent in the study by Maior et al. to 96.58 percent for the UTA-RLDD and 91.60 percent for the NTHU-DDD. This shows how well ensemble methods work for dealing with complicated data structures.

Moreover, the CNN models displayed excellent advancements, with an accuracy of 99.92% for the UTA-RLDD compared to 96% in the study by Nasri et al. [33], demonstrating substantial improvements in DL for image classification. The YOLO models, namely YOLOv5 and YOLOv8, were distinguished by their exceptional accuracy rates, achieving 100% for the UTA-RLDD, an improvement over the 97.5% achieved in the work of Krishna et al. [35] and 96.9% achieved in the study by Xie et al. [71], respectively. However, YOLOv8 experienced a decline to 77.33% for the YawDD, likely due to dataset-specific challenges. On the other hand, Faster R-CNN, which achieved 90.5% accuracy in the study by Redd et al. [72], performed worse on most datasets, only achieving 82.95% accuracy for the UTA-RLDD, which shows its flaws.

### 4.5. Ethical Considerations

Drowsiness detection systems, based on facial analysis and ML techniques, introduce several ethical concerns, notably concerning the safeguarding of personal privacy. These systems necessitate the continuous monitoring and analysis of drivers’ facial expressions and other personal data, potentially resulting in privacy infringements if not appropriately managed. Ensuring robust security measures for data collection, storage, and processing is essential to protect against unauthorized access and data breaches.

Furthermore, the potential misuse of these sensitive data raises substantial concerns. It is imperative to establish explicit norms and restrictions concerning data access, usage, and purposes. Transparency regarding the operations of these systems, the data collected, and their utilization is also crucial to maintaining public trust. Additionally, the possibility of bias in drowsiness detection algorithms could lead to the discriminatory treatment of certain groups of drivers. We must meticulously assess and mitigate this risk through the extensive testing and validation of the systems across diverse populations.

## 5. Conclusions

This paper explored real-time DDD using ML and CV techniques, focusing on facial analysis. The research assessed the efficacy of several methods for identifying drowsy driver behavior by employing diverse public datasets. In the realm of ML, the KNNs emerged as the leading algorithm, with an accuracy of 98.89%, a precision of 99.27%, and an F1 score of 98.86% on the UTA-RLDD. Among the CV algorithms, YOLOv5 achieved exceptional results, including 100% precision, 100% recall, and an mAP of 99.5% at IoU = 0.5, also on the UTA-RLDD. These findings highlight the substantial advancements achieved in accurately and efficiently identifying driver weariness.

This study investigated various techniques and datasets to develop a real-time system for detecting driver drowsiness. Implementing modern algorithms significantly enhances traditional detection methods, which are typically slow and intrusive. These algorithms deliver rapid and accurate assessments of driver alertness. Despite these achievements, some challenges remain, particularly with datasets like the YawDD, which highlight the need for further refinement in handling diverse driving conditions and behaviors.

Future endeavors will aim to refine these detection systems by enhancing their generalization across diverse conditions, integrating multimodal data sources—including physiological indicators—and expanding their real-time processing capabilities. These enhancements aim to increase the practical utility of these technologies, enabling their successful use in real-world environments to improve road safety.

## Figures and Tables

**Table 1:** Related studies summary.

Approach	Ref.	Methods	Dataset	Accuracy
Facial Analysis-Based (Images)	[32]	2D CNN	DDD [50] and CK+ [51]	93%
	[33]	CNN	UTA-RLDD [52]	96%
	[34]	CNN and VGG16 models	DDD [50]	97%
	[35]	YOLOv5	UTA-RLDD [52]	97.4%
Eyes and Mouth -Based (Images)	[37]	CNN	YawDD [52]	93.83%
	[36]	CNN	YawDD [52]	93.6%, 94.5%
	[42]	InceptionV3	NTHUDDD [53]	98.5%
	[43]	Privacy-preserving federated transfer learning method (PFTL-DDD)	NTHUDDD [53] and YAWDD [52]	84% (NTHUDDD),86% (YawDD)
	[38]	CNN_BiLSTM	MRL Eye [54]	94%
	[39]	SVM and Bayesian classifiers	Private	96.4%
	[40]	CNN	NTHUDDD [53], YawDD [52], and EMOCDS	95.67%
	[41]	SVM, Naïve Bayes, Random Forest (RF), bagging, and ANN	Private	98%
Video Sequence-Based	[44]	MS-STAGCN	NTHUDDD [53]	92.4%
	[45]	CNN and RNN	YawDD [52]	96.6%
	[46]	Two-stream spatiotemporal graph convolutional network (2s-STGCN)	YawDD [52] and NTHUDDD [53]	93.4% (YawDD),92.7% (NTHUDDD)
	[47]	Hierarchical Temporal Deep Belief Network (HTDBN)	NTHUDDD [53]	84.82%
	[48]	LSTM, VGG-16, Inception-V3, and DenseNet	Private	98%
	[49]	ResNet-50	NTHUDDD [53]	86%

**Table 2:** ML models’ parameters.

Algorithm	Parameters and Optimization
K-Nearest Neighbors	n_neighbors=1 (optimized)
Support Vector Machine	Kernel = linear, *C* = 1
Decision Tree	Random state = 42
RF	n_estimators=100, random state = 42

**Table 3:** CNN architecture layers and parameters.

Layer Block	Parameters
Input	Input shape: (150, 150, 3)
Block 1: Conv2D(32, (3, 3), activation=’elu’) BatchNormalization Conv2D(64, (3, 3), activation=’elu’) MaxPool2D(pool_size=(3, 3))	32 filters, kernel: (3, 3) 64 filters, kernel: (3, 3) Pooling size: (3, 3)
Block 2: Conv2D(64, (3, 3), activation=’elu’, padding=’same’) BatchNormalization Conv2D(64, (3, 3), activation=’elu’, padding=’same’) Add([x, block_1_output])	64 filters, kernel: (3, 3), padding=’same’ Skip connection
Block 3: Conv2D(64, (3, 3), activation=’elu’, padding=’same’) BatchNormalization Conv2D(64, (3, 3), activation=’elu’, padding=’same’) Add([x, block_2_output])	64 filters, kernel: (3, 3), padding=’same’ Skip connection
Block 4: Conv2D(128, (3, 3), activation=’elu’) MaxPool2D(pool_size=(2, 2))	128 filters, kernel: (3, 3) Pooling size: (2, 2)
Final Layers: GlobalAveragePooling2D Dense(256, activation=’elu’) Dense(1, activation=’sigmoid’)	256 units 1 unit, activation=’sigmoid’

**Table 4:** Performance Comparison of classifiers across datasets (in %).

Metric (%)	NTHUDDD				YawDD				UTA-RLDD			
	KNNs	SVM	DT	RF	KNNs	SVM	DT	RF	KNNs	SVM	DT	RF
**Test Accuracy**	95.72	93.54	77.25	91.60	89.51	81.13	67.14	80.20	**98.89**	97.76	87.12	96.58
**Precision**	95.34	93.32	77.46	91.10	88.45	81.55	68.29	81.99	99.27	97.45	84.29	**99.58**
**Recall**	96.31	94.05	78.10	92.56	91.76	82.26	68.47	79.25	**98.12**	97.29	85.83	92.39
**F1 Score**	95.72	93.53	77.24	91.59	89.47	81.10	67.09	80.19	**98.86**	97.71	86.87	96.47
**AUC**	95.71	93.53	77.24	91.58	89.42	81.09	67.08	80.23	**98.79**	97.70	86.95	96.05

**Table 5:** Performance metrics of CNN across datasets.

Metric (%)	NTHUDDD	YawDD	UTA-RLDD
Training Accuracy	99.31	99.75	99.92
Test Accuracy	98.22	93.31	99.97
Precision	98.14	93.9	100
Recall	98.36	93.05	99.93
F1 Score	98.22	93.31	99.97
AUC	98	93.32	99.96

**Table 6:** Performance metrics of YOLOv5, YOLOv8, and Faster-RCNN techniques across datasets.

Model	Metric (%)	NTHUDDD	YawDD	UTA-RLDD
YOLOv5	Precision	**99.9**	90.6	**99.9**
	Recall	**100**	94.9	**100**
	mAP50	**99.5**	98	**99.5**
	mAP50-95	**97.9**	90.1	91.7
YOLOv8	Precision	**99.9**	88.2	**99.9**
	Recall	**100**	92.6	**100**
	mAP50	**99.5**	97.2	**99.5**
	mAP50-95	**98**	90.3	91.7
Faster-RCNN	Precision	63.4	53.7	**81.0**
	Recall	77.7	69	**84.9**

**Table 7:** Comparison of results with previous studies.

Ref.	Classifiers	Previous Results		Our Results		
		Accuracy	Dataset	NTHU-DDD	YAWDD	UTA-RLDD
[68]	KNNs	83%	Private	95.72%	89.51%	98.89%
[69]	SVM	94.9%	DROZY	93.54%	81.13%	97.76%
[70]	DT	65.41%	UTA-RLDD	77.25%	67.14%	87.12%
[69]	RF	82.3%	DROZY	91.60%	80.20%	96.58%
[33]	CNN	96%	UTA-RLDD	99.31%	99.75%	99.92%
[35]	YOLOv5	97.5%	UTA-RLDD	100%	81%	100%
[71]	YOLOv8	96.9%	UTA-RLDD	100%	77.33%	100%
[72]	Faster-RCNN	90.5%	Private	70.55%	61.35%	82.95%

## Data Availability

The original data presented in the study are openly available. The NTHUDDD dataset: https://www.kaggle.com/datasets/banudeep/nthuddd2, accessed on 11 November 2024; the YawDD: https://www.kaggle.com/datasets/enider/yawdd-dataset, accessed on 14 November 2024; and the UTA-RLDD: https://sites.google.com/view/utarldd/home, accessed on 14 November 2024.

## Abbreviations

The following abbreviations are used in this manuscript:

AUC	Area Under the Curve
BiLSTM	Bidirectional Long Short-Term Memory
CNN	Convolutional Neural Network
CK+	Cohn–Kanade dataset
CV	Computer Vision
DDD	Driver Drowsiness Detection
DL	Deep Learning
DTs	Decision Trees
FPN	Feature Pyramid Network
HOG	Histogram of Oriented Gradients
KNNs	K-Nearest Neighbors
ML	Machine Learning
MS-STAGCN	Multi-Scale Spatial–Temporal Attention Graph Convolutional Network
RF	Random Forest
RPN	Region Proposal Network
SVMs	Support Vector Machines
UTA-RLDD	UTA Real-Life Drowsiness Dataset
ViTs	Vision Transformers
YOLO	You Only Look Once
